# Epigenetic silencing of *DACH1* induces the invasion and metastasis of gastric cancer by activating TGF-β signalling

**DOI:** 10.1111/jcmm.12325

**Published:** 2014-06-09

**Authors:** Wenji Yan, Kongming Wu, James G Herman, Malcolm V Brock, Yusen Zhou, Youyong Lu, Zhiqian Zhang, Yunsheng Yang, Mingzhou Guo

**Affiliations:** a Institute of Digestive Diseases, Chinese PLA General HospitalBeijing, China; b Tongji Hospital, Tongji Medical College of Huazhong University of Science and TechnologyWuhan, China; c Oncology Center, Johns Hopkins UniversityBaltimore, MD, USA; d State Key Laboratory of Pathogen and Biosecurity, Beijing Institute of Microbiology and EpidemiologyBeijing, China; e Department of Cell Biology, Beijing Institute for Cancer Research, Peking University School of OncologyBeijing, China

**Keywords:** DACH1, DNA methylation, gastric cancer, invasion, metastasis, chemosensitive marker, docetaxel

## Abstract

Gastric cancer (GC) is the fourth most common malignancy in males and the fifth most common malignancy in females worldwide. DACH1 is frequently methylated in hepatic and colorectal cancer. To further understand the regulation and mechanism of *DACH1* in GC, eight GC cell lines, eight cases of normal gastric mucosa, 98 cases of primary GC and 50 cases of adjacent non-tumour tissues were examined. Methylation-specific PCR, western blot, transwell assay and xenograft mice were used in this study. Loss of DACH1 expression correlated with promoter region methylation in GC cells, and re-expression was induced by 5-Aza-2′-deoxyazacytidine. *DACH1* is methylated in 63.3% (62/98) of primary GC and 38% (19/50) of adjacent non-tumour tissues, while no methylation was found in normal gastric mucosa. Methylation of *DACH1* correlated with reduced expression of DACH1 (*P* < 0.01), late tumour stage (stage III/IV) (*P* < 0.01) and lymph node metastasis (*P* < 0.05). DACH1 expression inhibited epithelial–mesenchymal transition and metastasis by inhibiting transforming growth factor (TGF)-β signalling and suppressed GC cell proliferation through inducing G2/M phase arrest. The tumour size is smaller in DACH1-expressed BGC823 cell xenograft mice than in unexpressed group (*P* < 0.01). Restoration of DACH1 expression also sensitized GC cells to docetaxel. These studies suggest that *DACH1* is frequently methylated in human GC and expression of DACH1 was controlled by promoter region methylation. DACH1 suppresses GC proliferation, invasion and metastasis by inhibiting TGF-β signalling pathways both *in vitro* and *in vivo*. Epigenetic silencing DACH1 may induce GC cells' resistance to docetaxel.

## Introduction

Gastric cancer (GC) is one of the most common malignancies, ranking fourth in males and fifth in females, respectively, and is a leading cause of cancer-related death worldwide [1]. Lacking early detection markers and effective therapeutic strategies, GC is usually diagnosed in late stages. Most of GC patients die of recurrence and metastasis, with a poor 5-year survival [2,3]. Dysregulation of normal signalling pathways is part of the transformation process in malignancy. Transforming growth factor (TGF)-β signalling functions in several biological processes, including cell proliferation, differentiation, migration and apoptosis. It plays important but paradoxical roles in carcinogenesis and cancer progression. TGF-β signalling promotes the invasion and metastasis by induction of epithelial–mesenchymal transition (EMT) in the later stages of cancer [4–8]. The EMT phenotype is characterized by loss of epithelial marker (E-cadherin) and increasing of mesenchymal factor (vimentin) expression, during which epithelial cells acquire mesenchymal properties while losing cell–cell interactions and apicobasal polarity [9,10]. EMT plays an important role in tumour metastasis and invasion and TGF-β signalling was reported to promote GC invasion and metastasis [11–14].

*Dachshund homolog 1 (DACH1)*, a homologue of Drosophila dac in human, is located in chromosome 13q22. Dac protein contains two conserved domains: dachbox-N and dachbox-C, both of which are highly conserved from drosophila to humans. DACH1 is expressed extensively in normal tissues and loss of DACH1 expression was reported related to poor prognosis in breast, endometrial and prostate cancers [15–17]. DACH1 has been shown to inhibit TGF-β signalling in breast and colorectal cancer [18,19].

In this study, we examined whether epigenetic changes in *DACH1* occurred in GCs and explored the role of DACH1 in tumour growth, invasion, metastasis and chemosensitivity in human GC.

## Material and methods

### Primary human GC samples and cell lines

Ninety-eight cases of primary GC and eight cases of normal gastric mucosa were collected as fresh frozen tissue from Chinese PLA General Hospital. Gastric cancer was classified by TNM stage, including stage I (*N* = 4), II (*N* = 8), III (*N* = 26) and IV (*N* = 60). Among 98 cancer samples, 32 cases of paraffin blocks are available with matched adjacent tissue. Eight cases of normal gastric mucosa were collected by biopsy under endoscopy from non-cancer patients. All samples were collected under the approved guidelines of the Chinese PLA General Hospital's institutional review board.

Seven gastric cell lines (AGS, BGC823, SGC-7901, NCI-N87, NUGC3, MGC803 and MKN45) and one immortalized human gastric mucosal cell line GES-1 were previously established and maintained in DMEM medium (Invitrogen, Carlsbad, CA, USA) supplemented with 10% foetal bovine serum (FBS). The information of these cells was published in our previous articles [20,21]. Cells were passaged 1:3 once 80% confluence (∼10^6^ cells) was reached on a 75 cm^2^ culture flask (NEST Biotechnology, Jiangsu, China).

### 5-Aza-2′-deoxycytidine treatment, RNA isolation and semi-quantitative RT-PCR

Gastric cancer cell lines were split to low density (30% confluence) 12 hrs before treatment. Cells were treated with 5-aza-2′-deoxycytidine (5-AZA; Sigma-Aldrich, St. Louis, MO, USA) at a concentration of 2 or 3 μM (MKN45) in the growth medium, which was exchanged every 24 hrs for a total 96-hr treatment. At the end of treatment course, cells were collected and total RNA was isolated by Trizol reagent (Invitrogen, Shanghai, China). Semi-quantitative reverse transcription-PCR (RT-PCR) was performed as described previously [19].

### Bisulphite modification, methylation specific PCR (MSP) and bisulfite sequencing (BSSQ)

Genomic DNA from GC cell lines and GC tissue samples were prepared by proteinase-K method. MSP and BSSQ were performed as described previously [22,23]. MSP primers and BSSQ primers was designed according to genomic sequences around transcription start site in the CpG island of *DACH1* gene (NM_080759.4) promoter region and synthesized (BGI, Beijing, china) to detect unmethylated (U) and methylated (M) alleles [19].

### Immunohistochemistry staining

Immunohistochemistry staining (IHC) was performed in 32 cases of available matched cancer and adjacent non-cancerous tissue samples. The procedure was performed as described previously [19]. Anti-DACH1 with 1/500 dilution (Proteintech, Chicago, IL, USA), anti-E-cadherin with 1/50 dilution (Bioworld Technology, Beijing, China) and anti-vimentin, anti-MMP-2, anti-MMP-9 (Bioworld Technology) with 1/100 dilution were incubated overnight at 4°C. The staining intensity and extent of the staining area were graded according to the German semi-quantitative scoring system as described before [19]. Staining intensity of the nucleus, cytoplasm and/or membrane (no staining = 0; weak staining = 1; moderate staining = 2; strong staining = 3); extent of stained cells (0% = 0, 1–24% = 1, 25–49% = 2, 50–74% = 3, 75–100% = 4). The final immunoreactive score (0–12) was determined by multiplying intensity score to the extent of stained cells score.

### Plasmid construction

The expression vectors for DACH1 wild-type or mutant type (DS and ΔDS) were gift from Dr. Cvekl. Reporter constructs, including SBE-4 Luc, which contains Smad-binding elements, were described as previously [18]. *DACH1* was subcloned into plenti6-GFP lentivirus expression vector, and DACH1 expression lentiviral or empty vectors were packaged by using ViraPowerTM lentiviral expression system (Invitrogen) to infect BGC823 and AGS cell lines to establish stable expression cells. Lipofectamine 2000 (Invitrogen) was used for plasmid transfection. All constructs were confirmed by sequencing.

### DACH1 knocking down by siRNA

Four selected siRNAs targeting *DACH1* and RNAi Negative Control Duplex were used in this study. The sequences are as follows: siRNA duplex 1 (sense:5′-GCCUCCUAAGAGGACUCAATT-3′; anti-sense: 5′-UUGAGUCCUCUUAGGAGGCTT-3′); siRNA duplex 2 (sense: 5′-GCAGGAAGCACUUGAGUUUTT-3′; anti-sense: 5′-AAACUCAAGUGCUUCCUGCTT-3′). RNAi Negative Control Duplex (sense: 5′-UUCUCCGAACGUGUCACG UTT-3′; antisense: 5′-ACGUGACACGUUCGGAGAATT-3). RNAi oligonucleotide or RNAi Negative Control Duplex (GenePharma Co., Shanghai, China) was transfected into GES-1 cells according to the manufacturer's instructions. The siRNA duplex 1 is more effective.

### Dual-Luciferase reporter assay

BGC823 and AGS cells were seeded at 1 × 10^4^/well in 96-well plates and incubated for 24 hrs. The transfection was described previously [19]. Relative luciferase activities were measured with the Dual Luciferase Reporter Assay system (Promega, Shanghai, China) according to the manufacturer's protocol. For each experiment, the luciferase reporter assay was repeated three times.

### Transwell migration assay

The effect of DACH1 on cell migration was detected by using the COSTAR transwell (Corning Incorporated, Beijing, China). DACH1-unexpressed and -re-expressed BGC823 and AGS cells were harvested and suspended in the serum-free medium. Cell suspensions were then placed into the upper well at a concentration of 2 × 10^4^ cells/200 μl, while the complete medium with 10% FBS was placed into the lower well (500 μl). The chamber was incubated for 16 hrs. The cells still on the upper surface were scraped gently and washed out with PBS three times. The cells that migrated to the lower surface of the membrane were stained with crystal violet and counted in three independent high-power fields (×200).

### Cell invasion assay

2 × 10^4^ DACH1 -unexpressed and -re-expressed BGC823 and AGS cells were suspended in 200 μl of serum-free medium and loaded onto the upper compartment of an invasion chamber containing a polycarbonate membrane with an 8 μm pore size, which was coated with a layer of extracellular matrix (ECM; Matrigel™, BD, Beijing, China). After 24 hrs of incubation, the invasive cells that migrated through the ECM layer to the complete medium in the lower compartment were stained with crystal violet and the number of invaded cells was counted in three independent high-power fields (×200).

### Protein preparation and western blotting

Protein preparation and western blot were performed as described previously [19]. The antibodies for immune blot analysis were as follows: mouse anti-Flag M2 (Sigma-Aldrich), rabbit anti-DACH1 (Proteintech), rabbit cyclinB1 (Bioworld Technology) and rabbit cdc2 (Bioworld Technology), rabbit anti-phospho-SMAD3 (Cell Signaling Technology, Inc, Shanghai, China), rabbit anti-phospho-SMAD2 (Millipore, Billerica, MA, USA), rabbit polyclonal anti-SMAD3, anti-SMAD2, anti-E-cadherin, anti-vimentin, anti-MMP-2, anti-MMP-9 (Bioworld Technology). The bands were visualized by enhanced chemiluminescence (Pierce Bioscience, Shanghai, China).

### Colony formation and cell viability detection

DACH1 unexpressed and stably expressed cells (1000 cells/well) were plated in 2 ml complete growth medium. The medium and reagents were changed every 48 hrs. Two weeks later, cells were fixed with 75% ethanol for 30 min. and stained with 0.2% crystal violet (Beyotime, Nanjing, China) for 20 min. and counted. The experiment was repeated three times.

DACH1 unexpressed and stably expressed cells were seeded onto 96-well plates (3 × 10^3^ cells/well), cell viability was measured daily by the Cell Counting Kit-8 (CCK8 kit; Dojindo Laboratories, Gaithersburg, MD, USA) for 4 days following the instruction of manufacturer.

### Flow cytometry analysis

DACH1 -unexpressed and -re-expressed BGC823 and AGS cells were starved 12 hrs for synchronization, the cells were re-stimulated with 10% FBS for 24 hrs. The cells were treated by Cell Cycle Detection Kit (KeyGen Biotech, Nanjing, China) following the instruction of manufacturer and then sorted by FACS Calibur (BD Biosciences, Franklin Lakes, NJ, USA). The cell phase distribution was analysed by WinMDI v. 2.9 software (Scripps Research Institute, La Jolla, CA, USA).

### Chemosensitivity detection

DACH1 -unexpressed and -re-expressed BGC823 and AGS cells were treated by docetaxel for 48 hrs and then the cell viability was tested by CCK8 kit (Dojindo Laboratories). Different concentration of docetaxel was applied to treat BGC823 and AGS cells. The concentration of docetaxel for treatment of BGC823 cells was 0, 2.5, 10, 20, 40 and 160 μg/ml and the concentration of docetaxel for treatment of AGS cells was 0, 0.02, 0.2, 2, 10 and 100 μg/ml. The cell viability was evaluated by the equation: the percentage of viable cells (%) = [A_450-630_(treated) − A_450-630_(blank)]/[A_450-630_(control) − A_450-630_(blank)] × 100%. IC_50_ was defined as the concentration of docetaxel for 50% inhibition of cell growth.

### *In vivo* tumourigenicity

DACH1 unexpressed and stably expressed BGC823 cells (3 × 10^6^ cells in 0.2 ml phosphate-buffered saline) were inoculated subcutaneously into the dorsal right flank of 4-week-old male Balb/c nude mice (*n* = 7) respectively. Tumour volume was assessed every 2 days for 3 weeks since 5 days after implantation. Tumour volume was calculated according to the following formula: *V* = *L* × *W*^2^/2 where *V*, volume (mm^3^); *L*, biggest diameter (mm); *W*, smallest diameter (mm). All procedures were approved by the Animal Ethics Committee of the Chinese PLA General Hospital.

### Statistical Analysis

SPSS 15.0 software was employed and all data are presented as means ± SD of at least three independent experiments. Chi-squared test or Fisher's exact test was used to analyse the association of clinical factors and the methylation status and the association of DACH1 expression and methylation status. Student's *t*-test was employed to compare two means. Statistical differences are presented at probability levels of *P* < 0.05 and *P* < 0.01.

## Results

### DACH1 was silenced by promoter region hypermethylation in GC

**Fig. 1:**
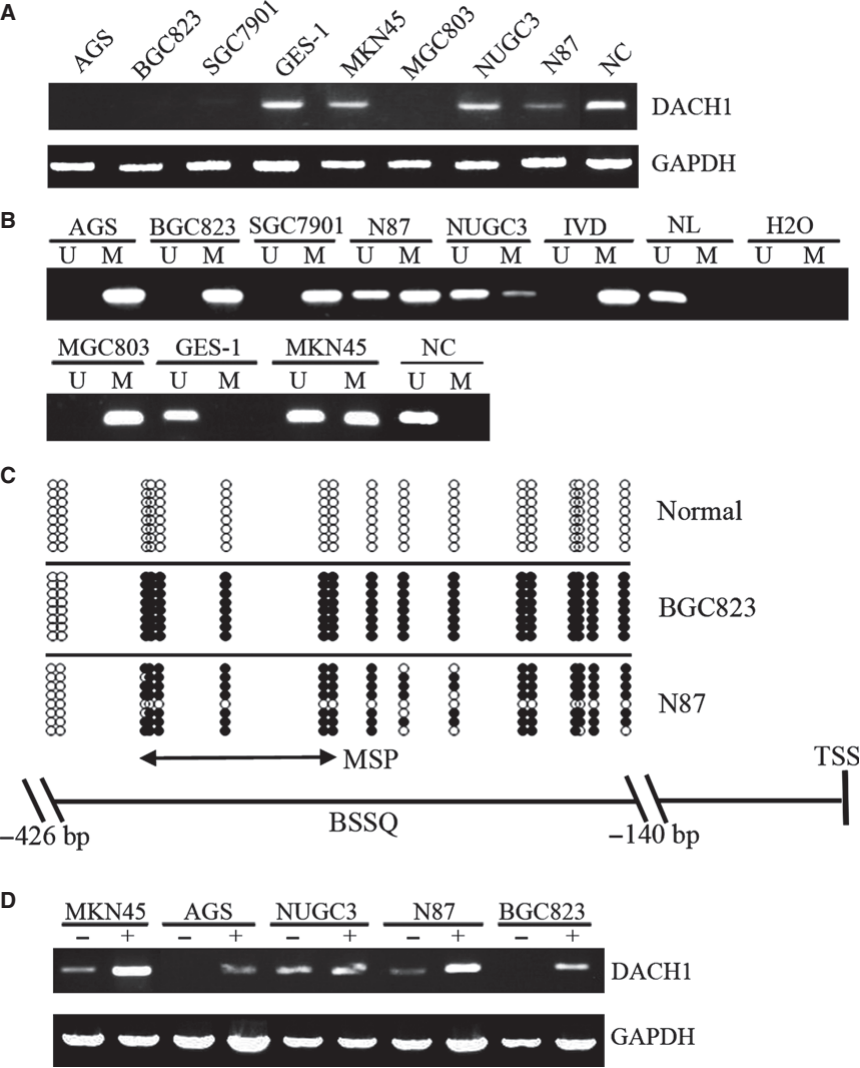
Down-regulation of DACH1 expression by DNA methylation in gastric cancer cell lines. (**A**) Expression of DACH1 was analysed by semi-quantitative RT-PCR in gastric cancer cell lines (BGC823, AGS, NCI-N87, MKN45, NUGC3, MGC803 and SGC7901), the immortalized human gastric mucosal cell line GES-1 and a case of normal control gastric mucosa (NC). GAPDH: Internal control of cDNA quality. (**B**) MSP results of *DACH1* in gastric cancer cell lines, GES-1 and normal control gastric mucosa (NC). IVD: *in vitro* methylated DNA serves as methylation positive control, NL: normal blood lymphocyte DNA, serves as unmethylation control. U: unmethylated alleles; M: methylated alleles. (**C**) Sodium bisulfite sequencing results of *DACH1* promoter region (−426 bp to −140 bp). BGC823 and NCI-N87: gastric cancer cell lines; Normal: normal gastric mucosa. Double-headed arrow: MSP product site, spanned 130 bp. Filled circles represent methylated CpG sites, and open circles denote unmethylated CpG sites. (**D**) Expression level of DACH1 detected by semi-quantitative RT-PCR; (−): absence of 5-Aza, (+): presence of 5-Aza.

*DACH1* was found frequently methylated in hepatocellular carcinoma and colorectal cancer in our previous study [19,24]. To determine whether this change occurred in GC, we first analysed epigenetic changes and DACH1 expression in seven human GC cell lines, one immortalized normal gastric epithelia cell (GES-1) and one case of normal gastric mucosa. Loss of DACH1 expression and complete methylation was found in BGC823, AGS, SGC7901 and MGC803 cells. Partial methylation was found in NCI-N87, MKN45 and NUGC3 cells, and reduced expression was found in NCI-N87 cells. The normal gastric epithelial cell line GES-1 and the normal gastric mucosa were found to be completely unmethylated and to express DACH1 (Fig. 1A and B). To further validate the specificity of MSP primers and the methylation density in promoter region, BSSQ was performed (Fig. 1C). Dense methylation was found in BGC823 cells, partial methylation was detected in NCI-N87 and a completely unmethylated promoter was found in normal gastric mucosa, confirming the findings obtained by using MSP. Loss of DACH1 expression correlated with promoter region hypermethylation. To confirm that DACH1 expression was directly regulated by promoter region methylation, MKN45, AGS, NUGC3, N87 and BGC823 cells were treated with the demethylating agent 5-AZA. Re-expression of DACH1 was found in BGC823 and AGS cell lines (originally without expression) and increased expression was found in NCI-N87 and MKN45 cell lines (partially methylated), while no expression change was found in NUGC3, which already expresses DACH1 (Fig. 1D). These results suggest that DACH1 expression was repressed by promoter region hypermethylation in most of the GC cell lines.

### *DACH1* was frequently methylated in human primary GC and promoter region hypermethylation was associated with reduction of DACH1 expression

**Fig. 2:**
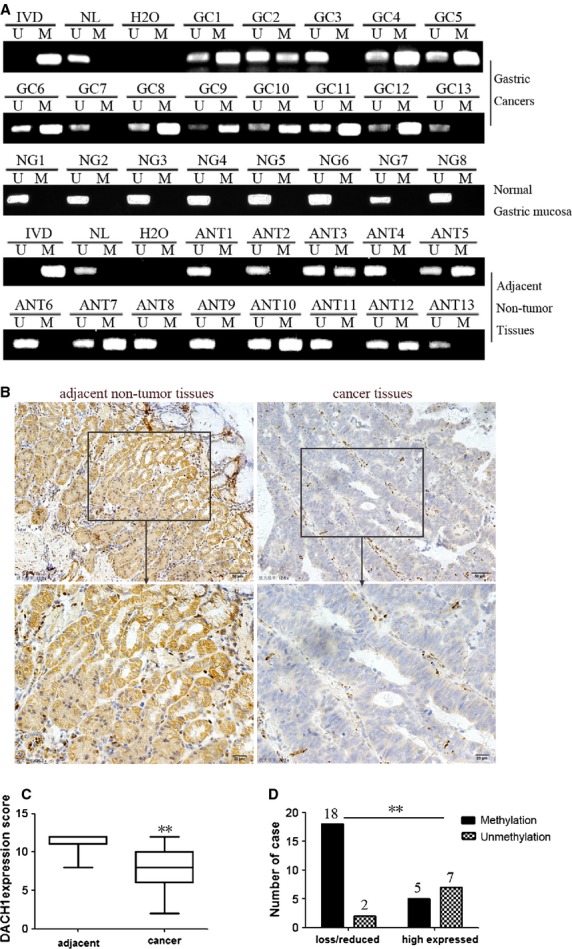
DACH1 expression and methylation status in primary gastric cancer. (**A**) Representative MSP results of *DACH1* methylation status in gastric cancer tissues (GC), normal gastric mucosa (NG) and adjacent non-cancerous tissues (ANT). (**B**) Representative images of DACH1 protein expression in gastric cancer tissues and their adjacent non-tumour tissues determined by immunohistochemistry (IHC). (up, ×200; below, ×400). (**C**) DACH1 expression levels are shown as box plots, the horizontal lines represent the median score; the bottom and top of the boxes representing the 25th and 75th percentiles respectively; and the vertical bars representing the range of expression level (**P < 0.01). (**D**) The correlation of DNA methylation and loss/reduced DACH1 expression in 32 available matched primary GCs (**P < 0.01).

To examine DNA methylation of *DACH1* in GC, 98 cases of primary GC samples, eight cases of normal gastric mucosa and 50 cases of adjacent non-tumour tissues were examined by MSP (Fig. 2A). Methylation was found in 63.3% (62/98) GC and 38% (19/50) adjacent non-tumour tissues. This methylation frequency was significantly higher in GC samples than in adjacent non-tumour tissues (*P* < 0.01), and no methylation was found in normal gastric mucosa from patients without cancer. The presence of methylation in some cases of adjacent normal appearing tissue suggests that DACH1 methylation is an early event of GC. As shown in Table 1, *DACH1* methylation was significantly associated with late tumour stage (stage III/IV; *P* < 0.01) and lymph node metastasis (*P* < 0.05), but no association was found between *DACH1* methylation and age, gender, differentiation, intravascular cancerous emboli or tumour size.

**Table 1:** Clinicopathological characteristics and methylation status of patients with gastric cancer (*n* = 98)

		Methylation status		
Clinical parameter	No.	Methylated *n* = 62 (63.3%)	Unmethylated *n* = 36 (36.7%)	*P*†-value
Age (year)				
<50	21	15 (71.4%)	6 (28.6%)	0.3813
≥50	77	47 (61%)	30 (39%)	
Gender				
Male	72	46 (63.9%)	26 (36.1%)	0.8313
Female	26	16 (61.5%)	10 (38.5%)	
Differentiation				
Poorly	74	50 (67.6%)	24 (32.4%)	0.1208
Moderately/Well	24	12 (50%)	12 (50%)	
Tumour stage				
I/II	12	3 (25%)	9 (75%)	0.0089**
III/IV	86	59 (68.6%)	27 (31.4%)	
Lymph node metastasis				
Negative	12	4 (33.3%)	8 (66.7%)	0.0481*
Positive	86	58 (67.4%)	28 (32.6%)	
Intravascular cancerous emboli				
Negative	58	34 (58.6%)	24 (41.4%)	0.2508
Positive	40	28 (70%)	12 (30%)	
Tumour size				
≥5 cm	64	44 (68.8%)	20 (31.2%)	0.1223
<5 cm	34	18 (52.9%)	16 (47.1%)	

† *P*-values are obtained from chi-squared test. Statistically significant,

* *P* < 0.05;

** *P* < 0.01.

The association of promoter region hypermethylation and DACH1 expression was analysed in 32 cases with available matched GC and adjacent tissue samples. DACH1 staining was observed in both the nucleus and cytoplasm. The expression of DACH1 was reduced significantly in cancer tissue compared with the adjacent tissue (*P* < 0.01, Fig. 2B and C). Among 20 cancer cases with lost/reduced DACH1 expression, 18 cases were methylated (90%). In contrast, among 12 cases of DACH1-expressed cancer tissue samples, only five cases were methylated (41.67%). Loss/reduction of DACH1 expression was significantly associated with promoter region hypermethylation in the tumours (*P* < 0.01, Fig. 2D). Despite the presence of methylation in some adjacent normal tissues, DACH1 expression was observed in the majority of normal appearing cells, suggesting that DACH1 methylation, detected with the sensitive MSP approach, was present in the minority of normal appearing cells and clonal progression, with methylation present in all cells within transformed tissues, was not yet present in these normal areas. Our results comparing DNA methylation with protein expression suggest that DACH1 expression is repressed by promoter region hypermethylation in most primary GCs.

### Restoration of DACH1 expression inhibits TGF-β signalling in GC

**Fig. 3:**
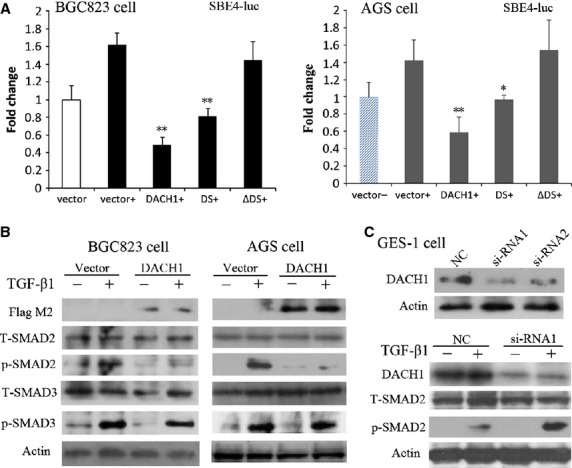
Effect of DACH1 on TGF-β signalling. (**A**) Luciferase activity was detected in BGC823 and AGS cells transfected with empty vector, DACH1 (200 ng/well), DS (40 ng/well) or ΔDS (160 ng/well), with 10 ng/ml TGF-β1 addition in each group (*P < 0.05, **P < 0.01). (**B**) Expression of TGF-β signalling downstream genes in DACH1 -unexpressed and -re-expressed BGC823 and AGS cells detected by Western blot; (−): absence of TGF-β1, (+): presence of TGF-β1 (T-: total; p-: phosphorylated). (**C**) The level of T-SMAD2 and p-SMAD2 detected by Western blot before and after knocking down DACH1 in GES-1 cells.

DACH1 protein contains two domains: DachBox-N and DachBox-C. Human DachBox-N shares approximately 28% amino acid identity with the SKI/SNO proteins, inhibitors of TGF-β signalling. Thus, the DachBox-N domain is also known as SKI/SNO (DS) domain [25,26]. TGF-β signalling was inhibited by DACH1 in breast and colorectal cancer by DS domain [18,19]. The function of the full-length DACH1, DS (DACH1 fragment, which encodes Smad-binding proteins' domain) and ΔDS (fragment of DACH1 deleted DS) was analysed by Dual-Luciferase reporter assay. SBE4 luciferase activity was inhibited by full-length DACH1 and DS significantly in both BGC823 and AGS cells (*P* < 0.05, Fig. 3A), but the activity of SBE4 has no changes before and after ΔDS transfection (*P* > 0.05). The results demonstrate that DS domain is critical for DACH1 function in TGF-β signalling in GC.

To further characterize the role of DACH1 in TGF-β signalling, phosphorylated SMAD2 and phosphorylated SMAD3 were examined by western blot in DACH1 -unexpressed and -re-expressed BGC823 and AGS cells. The TGF-β induced phosphorylation of SMAD2 was reduced in DACH1 expressing cells, while no change was found in phosphorylated SMAD3 after restoration of DACH1 expression (Fig. 3B). The inhibition function in TGF-β signalling was further confirmed by knocking down DACH1 in GES-1 cells (Fig. 3C) where DACH1 knock-down was associated with an increase in TGF-β-induced phosphorylation of SMAD2. These results suggest that DACH1 inhibits TGF-β signalling in GC through inhibition of SMAD2 phosphorylation.

### DACH1 suppresses cell invasion and migration by inhibiting EMT caused by TGF-β signalling in GC

**Fig. 4:**
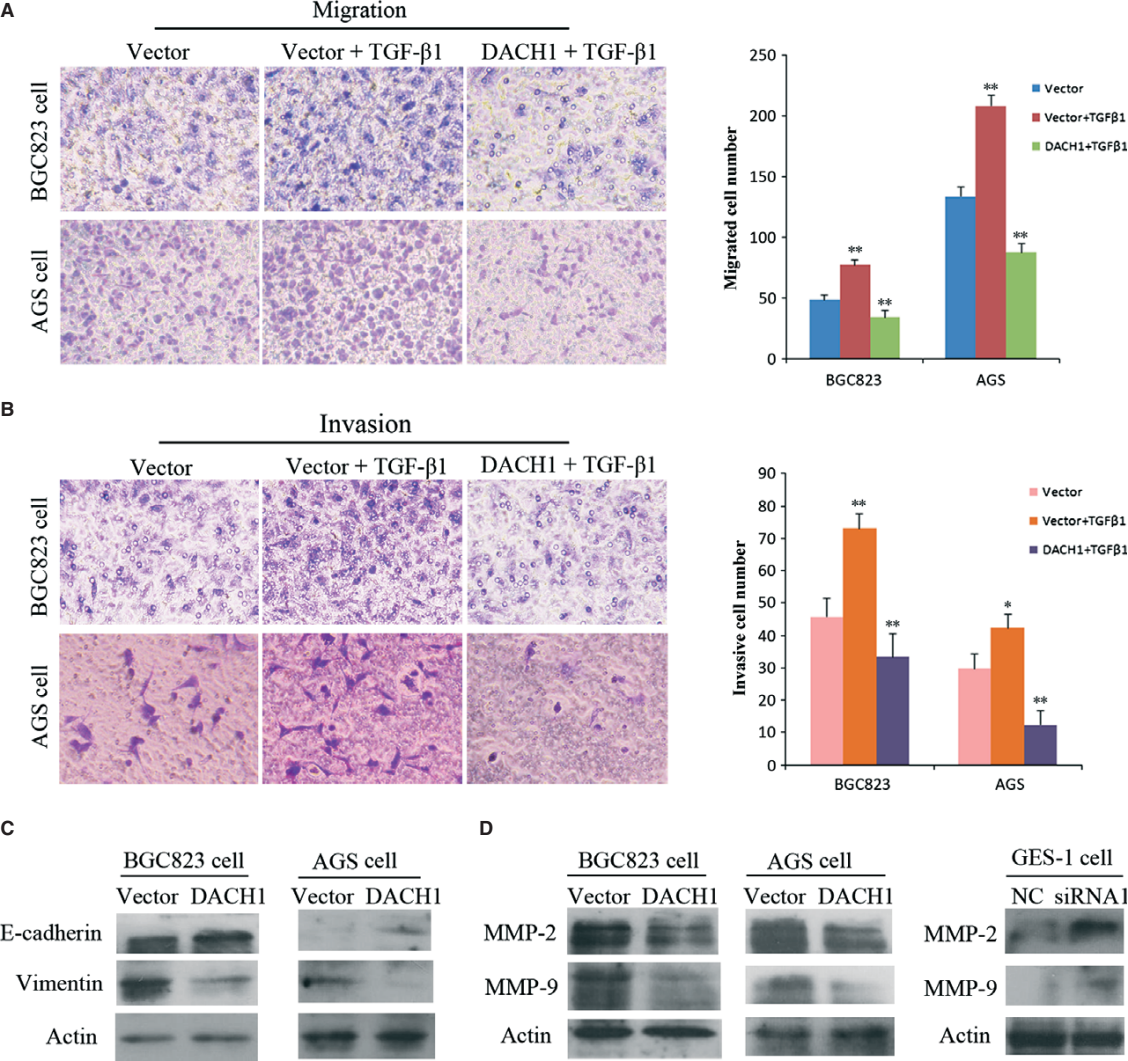
The effect of DACH1 on gastric cancer EMT and metastasis. (**A**) Trans-well assay results: the number of migrated cells' in empty vector group, empty vector with TGF-β1 addition group and re-expression of DACH1 plus addition of TGF-β1 group (*P < 0.05, **P < 0.01). (**B**) Invasion assay results: the number of invasive cells' in empty vector group, empty vector with TGF-β1 addition group and re-expression of DACH1 plus addition of TGF-β1 group (*P < 0.05, **P < 0.01). (**C**) The expression of E-cadherin and vimentin detected by Western blot in DACH1 -unexpressed and -re-expressed BGC823 and AGS cells. (**D**) The left panel shows the expression of MMP-2 and MMP-9 tested by Western blot in DACH1 -unexpressed and -re-expressed BGC823/AGS cells; the right panel shows the expression of MMP-2 and MMP-9 in GES-1 cells before and after DACH1 knocking down.

Epithelial–mesenchymal transition is related to cancer invasion and metastasis [27,28]. TGF-β1 may induce EMT by down-regulating E-cadherin and up-regulating vimentin in GC cells [29]. To see the role of DACH1 in cell migration and invasion, a transwell assay was carried out with BGC823 and AGS cells. The number of migrated BGC823 cells under each field of microscope was 48.67 ± 4.04, 77.67 ± 3.51 and 34.33 ± 5.86 in empty vector group, empty vector adding TGF-β1 group and re-expression of DACH1 adding TGF-β1 group respectively. The number of migrated AGS cells under each microscope was 134 ± 7.55, 208.67 ± 8.14 and 87.67 ± 7.02 in empty vector group, empty vector adding TGF-β1 group and re-expression of DACH1 adding TGF-β1 group respectively. Thus, an increase in migrating cells was found with TGF-β1 treatment compared with empty vector (*P* < 0.01), but less migration cells were found when DACH1 expression was restored, even when cells also received TGF-β1 treatment (*P* < 0.01, Fig. 4A). The results suggest that cell migration was promoted by TGF-β1 and was suppressed by DACH1 in BGC823 and AGS cells. As shown in Figure 4B, similar results were obtained by using transwell matrigel experiment, which mimics invasion. TGF-β1 promotes cell invasion and DACH1 suppresses cell invasion in BGC823 and AGS cells (*P* < 0.01, Fig. 4B). To see the effect of DACH1 on EMT, E-cadherin and vimentin, EMT-related markers, were examined by western blot in DACH1 -unexpressed and -re-expressed BGC823 and AGS cells. Expression of E-cadherin was up-regulated and vimentin was down-regulated in DACH1-expressed BGC823 and AGS cells (Fig. 4C) compared with the non-expressing parental cell lines. As a family of zinc-containing endopeptidases, matrix metalloproteinases (MMPs) are the major enzymes involved in ECM degradation [30,31]. Two representative members, MMP-2 and MMP-9, were reported highly expressed in invasive tumours [32,33]. The expression of MMP-2 and MMP-9 was reduced after re-expression of DACH1 in GC cells, and this relationship was confirmed by knocking down DACH1 in GES-1 cells (Fig. 4D), which show an increase in MMP2 and MMP9 with reduction of DACH1 expression.

### Restoration of DACH1 expression inhibits GC cell proliferation and induces G2/M arrest

**Fig. 5:**
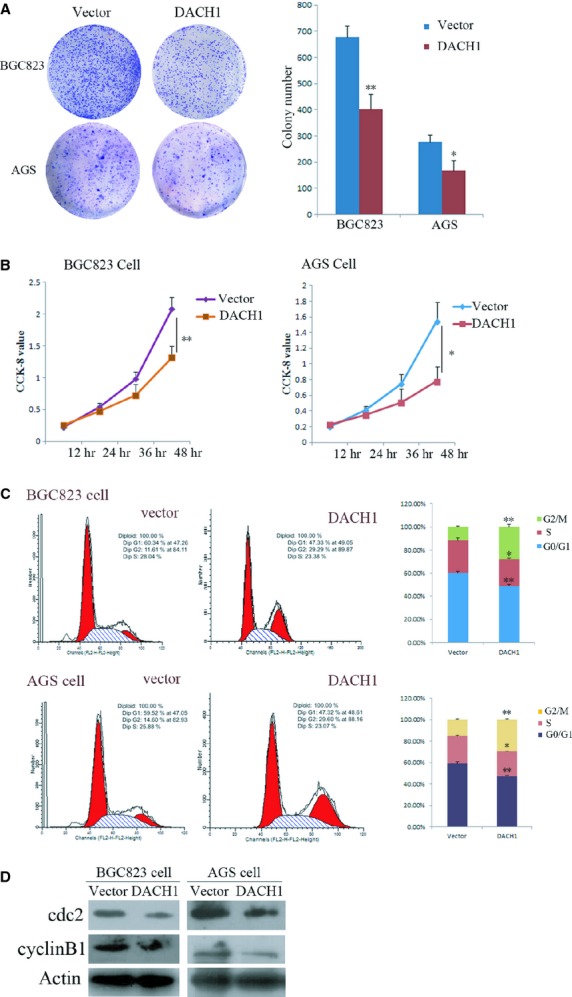
Effect of DACH1 on tumour growth and cell cycle in gastric cancer cells. (**A**) Colony formation results of DACH1 -unexpressed and -re-expressed gastric cancer cells. The left panel: the representative colony formation results of DACH1 -unexpressed and -re-expressed BGC823 and AGS cells. Right panel: colony numbers (*P < 0.05, **P < 0.01). (**B**) Growth curves: cell viability was tested by CCK-8 kit in BGC823 and AGS cells (*P < 0.05, **P < 0.01). (**C**) Representative cell cycle and flow cytometry data (*P < 0.05, **P < 0.01). (**D**) The expression of cyclinB1 and cdc2 detected by Western blot in DACH1 -unexpressed and -re-expressed BGC823 and AGS cells.

To evaluate the effect of DACH1 on GC cell proliferation, colony formation and cell viability were examined. As shown in Figure 5A, the colony number was 678 ± 43 *versus* 402 ± 57 in BGC823 cells (*P* < 0.01) and 276 ± 28 *versus* 167 ± 39 in AGS (*P* < 0.05) cells before and after restoration of DACH1 expression, showing a 40% reduction in colony formation with restored DACH1 expression. Cell viability was determined by using the CCK-8 kit. The OD value was 1.55 ± 0.24 *versus* 0.78 ± 0.18 in BGC823 cells (*P* < 0.01) and 2.08 ± 0.19 *versus* 1.32 ± 0.17 in AGS cells (*P* < 0.05) before and after re-expression of DACH1 (Fig. 5B), with a reduction of viability by 37–50% after re-expression of DACH1 in GC cells. These results suggest that DACH1 inhibits GC cell proliferation. To examine the way in which this occurred through cell cycle alterations, flow cytometry technique was employed. The cell phase distribution of DACH1 -unexpressed and -re-expressed BGC823 cells was as follows: 60.22 ± 1.41% *versus* 48.85 ± 1.37% for G0/1 phase (*P* < 0.01), 28.17 ± 2.2% *versus* 23.33 ± 1.2% for S phase (*P* < 0.05) and 11.6 ± 0.8% *versus* 27.83 ± 2.05% for G2/M phase (*P* < 0.01). The cell phase distribution of DACH1 -unexpressed and -re-expressed AGS cells was as follows: 59.03 ± 1.65% *versus* 47.11 ± 1.12% for G0/1 phase (*P* < 0.01), 25.66 ± 0.68% *versus* 23.32 ± 0.33% for S phase (*P* < 0.05) and 15.31 ± 1.12% *versus* 29.53 ± 0.85% for G2/M phase (*P* < 0.01). For both cell lines, G0/1 and S phase were reduced and G2/M phase was increased significantly in BGC823 and AGS cells (Fig. 5C), suggesting induction of a G2/M arrest. To further validate the effect of DACH1 on cell cycle, the expression of cdc2 and cyclinB1, the mitosis initiators, was examined by western blot in BGC823 and AGS cells (Fig. 5D). Both cdc2 and cyclinB1 were decreased after re-expression of DACH1. These results further demonstrated the G2/M phase arrest induced by DACH1 in GC.

### DACH1 sensitizes GC cells to docetaxel

**Fig. 6:**
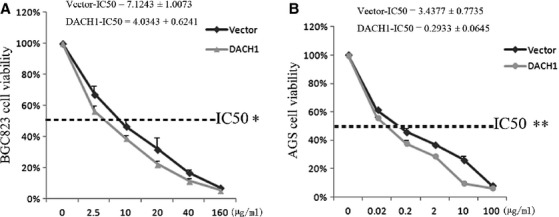
The chemosensitivity of DACH1 -unexpressed and -re-expressed BGC823 and AGS cells to docetaxel. (**A**) The responsive curves of DACH1 -unexpressed and -re-expressed BGC823 cells in different concentration of docetaxel (0, 2.5, 10, 20, 40, 160 μg/ml). (**B**) The dose–response curves of DACH1 -unexpressed and -re-expressed AGS cells in different concentrations of docetaxel (0, 0.02, 0.2, 2, 10, 100 μg/ml). The viability of cells was measured by CCK-8 after docetaxel treatment for 48 hrs.

G2/M arrest was induced by DACH1 in BGC823 and AGS cells. Docetaxel is a cytotoxic reagent that causes microtubule poly-merization-stabilization by binding to the tubulin. This, then suppresses dynamic properties at the mitotic spindle and induces cancer cell mitotic arrest (G2/M arrest) and apoptosis. To determine whether there was an interaction between DACH1 expression and docetaxel, cell viability was analysed by CCK-8 kit in DACH1 unexpressed and expressed GC cells. As shown in Figure 6, the IC50 of docetaxel in DACH1 -unexpressed and -re-expressed BGC823 cells was 4.0343 ± 0.6241 *versus* 7.1243 ± 1.0073 (*P* < 0.05, Fig. 6A). The IC50 of docetaxel in DACH1 -unexpressed and -re-expressed AGS cells was 0.2933 ± 0.0645 *versus* 3.4377 ± 0.7735 (*P* < 0.01, Fig. 6B). The results suggest that restored expression of DACH1 sensitized GC cells to docetaxel.

### DACH1 suppresses GC growth in xenograft mice

**Fig. 7:**
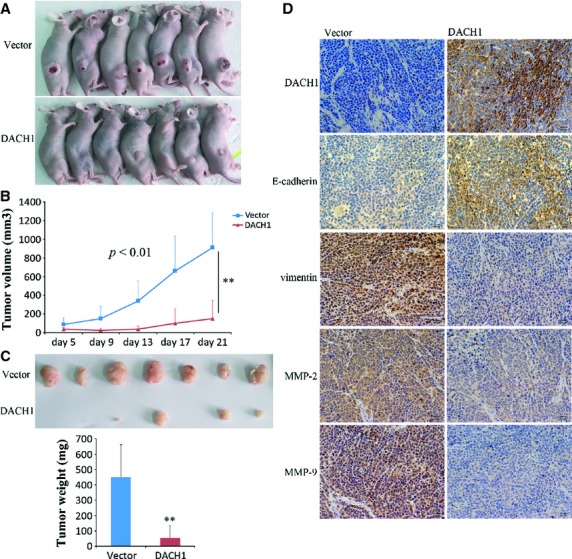
The effect of DACH1 on gastric cancer cell xenograft. (**A**) Representative picture of xenograft tumour of DACH1 -unexpressed (up) and -re-expressed BGC823 cells (low). (**B**) Subcutaneous tumour growth curve in xenograft mice with or without DACH1 re-expression (**P < 0.01). (**C**) Histogram represents average weight of xenograft in DACH1 -unexpressed and -re-expressed groups (**P < 0.01). (**D**) Immunohistochemical (IHC) staining in xenograft (×400).

To further explore the effect of DACH1 on GC growth *in vivo*, BGC823 cells with and without DACH1 expression were grown as xenograft in immune-compromised mice. As shown in Figure 7, the tumour size is smaller in DACH1 expressing BGC823 cell xenografts than in the parental non-expressing BGC823 cell xenografts (150.174 ± 191.064 mm^3^ *versus* 912.674 ± 373.444 mm^3^; *P* < 0.01, Fig. 7A and B), confirmed by measuring tumour weight, which is less in DACH1 expressing BGC823 cell xenografts than in parental BGC823 cell xenografts (54.29 ± 79.76 mg *versus* 450 ± 210 mg, *P* < 0.01, Fig. 7C). The expression of DACH1, E-cadherin, vimentin, MMP-2 and MMP-9 in xenografts was confirmed by IHC staining. Increased expression of E-cadherin and reduced expression of vimentin, MMP-2 and MMP-9 were revealed in DACH1-stably expressed BGC823 cell xenograft (Fig. 7D). These results suggest that DACH1 suppresses GC cell growth in xenograft mice, confirming the effects seen *in vitro*.

## Discussion

The expression of DACH1 has been reported to be reduced in different types of cancers, but increased in ovarian cancer [16,18,19,24,34,35]. We demonstrate here that DACH1 was frequently methylated in human GC and the expression of DACH1 was suppressed by promoter region methylation. *DACH1* methylation is associated with higher tumour stage and lymph node metastasis. These results suggest that methylation of *DACH1* may serve as a potential GC detection and prognostic marker. DACH1 suppresses GC growth both *in vitro* and *in vivo*, and further study found that DACH1 suppresses GC growth by inhibiting TGF-β signalling. It has been reported that TGF-β signalling suppresses tumour growth in the early stage and promotes the invasion and metastasis of cancer by induction of EMT in the later tumour stage [4–8]. Our study provides insight into this important but poorly understood switch. Aberrant activation of TGF-β signalling facilitates degradation of ECM, and degradation of the ECM is a necessity for invasion and dispersion of cancer cells and has been thoroughly studied during the last decade [36–38]. MMP-2 and MMP-9 are two major components of MMPs, which have been identified as key enzymes in this process. MMP-2 and MMP-9 can promote malignant cell progression and may facilitate the tumour growth, invasion and metastasis because of their ability to degrade type IV collagen of ECM and basement membrane [39–42]. MMP-2 promotes cleavage of ECM proteins and is intensively expressed by tumour and stromal components of cancer, while MMP-9 modulates permeability of vascular endothelium [42,43]. In our study, methylation of *DACH1* was associated with late tumour stage and lymph node metastasis. Cell migration and invasion were suppressed by DACH1 through inhibiting TGF-β signalling in BGC823 and AGS cells. The expression of E-cadherin was up-regulated and vimentin was down-regulated by DACH1 in BGC823 and AGS cells, and the expression of MMP-2 and MMP-9 was reduced by DACH1 through inhibiting TGF-β signalling. These results were validated by xenograft. Our results suggest that cell migration and invasion are suppressed by DACH1 through inhibiting EMT, which was induced by TGF-β signalling.

As DACH1 suppresses GC growth both *in vitro* and *in vivo*, it suggests that DACH1 is a tumour suppressor in human GC. Loss of DACH1 with progression of GC allows the growth-promoting activities of TGF-β to persist, while the invasive and growth inhibitory effects are lost. Cell cycle checkpoint dysfunction is often associated with sensitivity to chemotherapeutic agents. Microtubule-targeting drugs function in suppressing spindle microtubule dynamics, thus inhibiting the metaphase–anaphase, blocking mitosis and inducing apoptosis. Both docetaxel and paclitaxel are microtubule inhibitors. Paclitaxel suppresses spindle microtubule dynamics by allowing microtubule attachment, but altering the tension across the kinetochore in mitosis [44,45]. Docetaxel disrupted centrosome organization by affecting the late S phase, G2/M phase, which results in incomplete mitosis, accumulation of cells in the G2/M phase and cell death [46,47]. However, in GC, the response rate to docetaxel is 24%, with only partial responses and a median survival of 7.5 months [48]. As docetaxel is poorly tolerated clinically, it is important to find sensitive markers for personalized treatment. DACH1 was silenced by promoter region hypermethylation and G2/M phase arrest was induced by DACH1 in BGC823 and AGS cells. To explore the possible connection of DACH1 expression and docetaxel sensitivity, we detected the IC50 of docetaxel in DACH1 unexpressed and re-expressed BGC823 and AGS cells. Our results suggest that re-expression of DACH1 sensitized BGC823 and AGS cells to docetaxel. It suggests that DACH1 may enhance docetaxel to exert its cytotoxic activity through stabilizing microtubule assembly, leading to cell cycle arrest at G2/M phase. Another report demonstrated that enhancing acetylation of P53 could have potential implication for increasing the sensitivity of cancer cells to Taxol [49]. DACH1 was identified as a novel P53 binding partner [50,51]. Further study is needed to understand if DACH1 sensitizes GC cells to docetaxel through P53. Our results suggest that DACH1 methylation may serve as a docetaxel-resistant marker in GC and epigenetic therapy that reverses DACH1 silencing might result in sensitization to doecetaxel.

In conclusion, *DACH1* is frequently methylated in human GC and the expression of DACH1 was silenced by promoter region hypermethylation. *DACH1* methylation is associated with late tumour stage and lymph node metastasis. DACH1 suppresses GC proliferation, invasion and metastasis by inhibiting TGF-β signalling pathways both *in vitro* and *in vivo*. DACH1 re-expression sensitized GC cells to docetaxel.
